# Use of Novel High-Protein Functional Food Products as Part of a Calorie-Restricted Diet to Reduce Insulin Resistance and Increase Lean Body Mass in Adults: A Randomized Controlled Trial

**DOI:** 10.3390/nu9111182

**Published:** 2017-10-28

**Authors:** Carol S. Johnston, Barry Sears, Mary Perry, Jessica R. Knurick

**Affiliations:** 1 School of Nutrition and Health Promotion, Arizona State University, Tempe, AZ 85004, USA; jessrk87@gmail.com; 2 Inflammation Research Foundation, Peabody, MA 01960, USA; bsears@drsears.com (B.S.); mperry@zoneliving.com (M.P.)

**Keywords:** insulin resistance, calorie restriction, high-protein diet

## Abstract

Significant reductions in insulin resistance (IR) can be achieved by either calorie restriction or by the increase of lean mass. However, calorie restriction usually results in significant loss of lean mass. A 6-week randomized controlled feeding trial was conducted to determine if a calorie-restricted, high-protein diet (~125 g protein/day consumed evenly throughout the day) using novel functional foods would be more successful for reducing IR in comparison to a conventional diet (~80 g protein/day) with a similar level of calorie restriction. Healthy adults (age 20–75 years; body mass index, 20–42 kg/m^2^) with raised triglyceride/high-density lipoprotein ratios were randomly assigned to the control group (CON: test foods prepared using gluten-free commercial pasta and cereal) or to the high-protein group (HPR: test foods prepared using novel high-protein pasta and cereal both rich in wheat gluten). Mean weight loss did not differ between groups (−2.7 ± 2.6 and −3.2 ± 3.0 kg for CON (*n* = 11) and HPR (*n* = 10) respectively, *p* = 0.801); however, the 6-week change in fat-free mass (FFM) differed significantly between groups (−0.5 ± 1.5 and +1.5 ± 3.8 kg for CON and HPR respectively, *p* = 0.008). IR improved in HPR vs. CON participants (homeostasis model assessment-estimated insulin resistance [HOMAIR] change: −1.7 ± 1.4 and −0.7 ± 0.7 respectively; *p* = 0.020). The change in HOMA-IR was related to the change in FFM among participants (*r* = −0.511, *p* = 0.021). Thus, a high-protein diet using novel functional foods combined with modest calorie restriction was 140% more effective for reducing HOMA-IR in healthy adults compared to a lower protein, standard diet with an equal level of calorie restriction.

## 1. Introduction

Insulin resistance (IR) is characterized by the decreased ability of insulin to exert its metabolic actions on target cells. The molecular mechanisms of this failure are complex, but appear to be intimately linked with increased inflammation [1,2,3]. Significant reductions in IR can be achieved by calorie restriction and/or exercise [4,5]. In fact, strict calorie restriction (1100 calories per day) reduced IR in obese patients with or without type 2 diabetes by approximately 30% after only four days before any substantial weight loss had taken place [6]. Of the macronutrient changes, only reduced carbohydrate intake was significantly associated with reductions in fasting glucose concentrations in this trial suggesting an immediate physiological response to dietary carbohydrate restriction. However, a recent study in individuals with type 2 diabetes demonstrated that 12 weeks of significant calorie restriction (1450 calories per day) and enhanced exercise reduced IR by nearly 40% regardless of the macronutrient composition of the diet [7]. Yet, when calorie intake increased to 1700 calories per day for weight maintenance (weeks 13 to 24), there were no further reductions in IR even with continued enhanced exercise.

IR is also modified by a change in lean mass relative to total mass in the absence of weight loss, and retention of lean mass over time predicts reductions in IR [8]. It is well documented that calorie restriction is usually accompanied by reductions in both lean and fat mass, approximately 25% and 75% of total mass respectively [9]. However, high-protein diets can support the maintenance of lean mass, especially when energy is restricted for weight loss [9,10]. To achieve an anabolic response during calorie restriction, recent evidence suggests the importance of distributing adequate amounts of protein (~20 g of high-quality protein such as whey) in 3–4 meals over the course of the day to raise muscle intracellular amino acid concentrations and muscle protein synthesis via mRNA translation [11].

Hence, a calorie-restricted high-protein diet, with protein distributed evenly throughout the day may represent a particularly effective strategy for reducing IR under conditions of calorie restriction. This single-blinded, randomized controlled weight loss trial compared the efficacy of a standard, calorie-restricted control diet versus a protein-rich, calorie-restricted diet containing novel functional food products consumed at each meal daily, for reducing homeostasis model assessment-estimated insulin resistance (HOMA-IR) while simultaneously increasing fat-free mass (FFM) in adults at risk for IR.

## 2. Materials and Methods

### 2.1. Test Foods

High-quality protein pasta (orzo and fusilli) enriched with whey protein isolate, wheat gluten and egg white protein (% energy: 43/27/30 for carbohydrate/protein/fat) and high-protein flaked breakfast cereal enriched with wheat gluten (% energy: 35/30/35 for carbohydrate/protein/fat) were provided by Zone Labs, Inc. (Peabody, MA, USA). Commercial gluten-free pasta (Barilla, Northbrook, IL, USA; % energy: 63/13/24/ for carbohydrate/protein/fat) and commercial flaked cereal (Post, St. Louis, MO, USA; % energy: 76/6/18/ for carbohydrate/protein/fat) were used as controls.

### 2.2. Subjects

Healthy adults by self-report (age 20–75 years; body mass index (BMI) 20–42 kg/m^2^) were recruited from a campus community in the Phoenix metropolitan area via online advertisements and word of mouth in January 2015. Interested individuals were invited to complete an online survey to screen for history of food allergies or diet restrictions (e.g., vegetarian, gluten or lactose intolerant), insulin use, cigarette use, active disease states including diagnosed diabetes, anticipated changes to diet or physical activity levels, recent weight gain or loss (±10 pounds), and current or recent pregnancy or lactation. Prescription medication use by participants, including statins and hypertensive medications, was allowed if use had been consistent for the previous 3 months and would remain consistent during the trial. Volunteers were advised that the diet protocol was energy restricted to promote ~1 pound weight loss per week and that study foods (cereal and pasta dishes) would be provided for participants as their primary foods for the duration of the trial. Qualifying individuals were invited to the study site to discuss the food trial in detail and provide written informed consent. The Institutional Review Board at Arizona State University approved the study. The study was registered at ClinicalTrials.gov (identifier: NCT02851498).

### 2.3. Study Design

A fasting blood sample was collected from 92 consented adults (13 males) to determine the triglyceride/high-density lipoprotein (TG/HDL) ratio, an indicator of IR [12]. Those at or above the 50th percentile (>2.2 and >1.5 for the men and women respectively) were invited to participate in the feeding trial (*n* = 49). Thirty-nine participants agreed to start the trial (January and February 2015) and were stratified by age, BMI, and TG/HDL ratio and randomly assigned by coin toss to one of two groups: Control (CON, *n* = 19; test foods prepared using gluten-free commercial pasta and cereal) or high-protein (HPR, *n* = 20; test foods prepared using novel high-protein pasta and cereal rich in wheat gluten). Participants were scheduled for weekly visits during the 6-week study to weigh-in and pick up food packages. CON and HPR test foods were similar in appearance and taste, and participants were blinded to the diet assignment. All participants received individual instruction on the diet protocol (e.g., to consume the test cereal at breakfast and the pasta meals at lunch and dinner) and received personal diet plans for additional foods to be consumed in addition to the test foods. Individualized diet plans, designed to reduce total energy intake by 500 kcal below maintenance needs based on the Mifflin St Jeor equation, utilized an exchange system to promote healthy food choices for both groups in addition to the provided cereal and pasta dishes. Participants were counselled to eat low-fat dairy and meats as well as fruit and vegetables daily. The provided pasta meals followed a 7-day rotating menu. The participants received at no cost 14 different pasta dishes weekly during the study (e.g., seven lunch and seven dinner meals) along with seven prepackaged cereal aliquots. The fourteen pasta dishes were prepared weekly in the metabolic kitchen at Arizona State University and frozen prior to pick-up. The study foods provided 935 to 954 kcal on average each day. Table 1 displays the nutritional information for the CON and HPR cereals and pasta meals.

Participants recorded study compliance (daily cereal and pasta ingestion) on a calendar and recorded food intake daily in a written journal. A fasting venous blood sample was collected at trial weeks 0 and 6, and body weight and composition was determined using a bioelectrical impedance scale (Tanita, Arlington Heights, IL, USA) at these same time points using a standardized measurement protocol. Participants were asked to avoid moderate to heavy exercise on the day prior to blood collection and to fast overnight (no food or beverage with the exception of water for 10 h). The Profile of Mood States (POMS) questionnaire was also completed at trial weeks 0, and 6, and a physical activity questionnaire was completed at trial weeks 0, 3, and 6.

### 2.4. Blood Analyses

Fasting blood samples were analyzed in duplicate for serum glucose and secondary outcomes (total cholesterol, HDL cholesterol, low-density lipoprotein (LDL) cholesterol, and high-sensitivity C-reactive protein [hsCRP]) using a point-of-care COBAS C111 chemistry random access autoanalyzer (Roche Diagnostics, Indianapolis, IN, USA). Insulin concentrations were measured by radioimmunoassay (Millipore, St. Charles, MO, USA), and HOMA-IR was calculated as (fasting insulin μU/mL × fasting glucose mg/dL)/405. Other secondary outcomes included total antioxidant capacity, measured using a commercially available kit according to the manufacturer’s instructions (Cat No. 709001; Cayman Chemical, Ann Arbor, MI, USA), and fasting plasma concentrations of thiobarbituric acid reactive substances (TBARS), measured using a kit (Cat No. 0801192; Zeptometrix Corporation, Buffalo, NY, USA) and quantitated as malondialdehyde which reacts with TBARS acid to form a product that is measured spectrophotometrically. In addition, glucagon-like peptide-1 (GLP-1) and peptide YY (PYY) were analyzed in an aliquot of blood that was immediately stabilized by the addition of aprotinin and the DPPV inhibitor, and GLP-1 and PYY were later measured by commercial kits according to manufacturers’ instructions (Millipore GLP-1 (Active) Cat# EGLP-35K and Millipore Human PYY (Total) cat# EZHPYYT66K; EMD Millipore Corporation, Billerica, MA, USA). A chemiluminescence ELISA assay kit was used to quantitate high-molecular weight (HMW)-adiponectin (Cat # 80-ADPHU-CHO1; ALPCO, Salem, NH, USA). 8-isoprostane was measured using a competitive ELISA assay system (Caymen Chemical Cat # 516351; Caymen Chemical Co., Ann Arbor, MI, USA).

### 2.5. Statistical Analysis 

Data are presented as the mean ± standard deviation (SD). Power calculations indicated that a total of 42 participants were necessary for this parallel-design study to detect a 2.0 unit difference in HOMA-IR [13], at an 80% probability and 0.05 significance level, and assuming a 40% attrition rate common to feeding trials [14]. General Linear Model analyses were used to assess differences between means and effect size (partial eta squared, η^2^_p_) controlling for confounding variables as indicated. Data were transformed to achieve normality prior to analyses if necessary. Analyses were performed using SPSS software, version 22 (2013, IBM-SPSS Inc. Armonk, NY, USA). A *p*-value < 0.05 was considered significant and a large effect size (η^2^_p_ > 0.140; see [15]) was considered suggestive of physiological relevance.

## 3. Results

Of the 39 participants randomized to treatment groups (19 CON; 20 HPR), eight did not return to the test site after the baseline visit, and an additional 10 participants withdrew prior to the end of the 6-week trial. These latter participants withdrew from the trial during weeks 0–3 (with the exception of one that withdrew during week 4) due to time conflicts and/or a waning desire to consume the study test foods each day. No adverse effects were reported during the study. Baseline age, body weight, BMI, and the TG/HDL ratio did not differ significantly between those who did not complete the trial (*n* = 18) and those that did (*n* = 21). Moreover, using intention-to-treat calculations (e.g., last observation carried forward), the 6-week weight loss for all participants randomized to a treatment group did not differ significantly between groups (−2.0 ± 1.8 and −2.3 ± 2.9 kg for the CON and HPR groups respectively; *p* = 0.715). Hence, data are presented only for the 21 participants that completed the trial; the baseline data for these participants (11 CON; 10 HPR) are displayed in Table 2. There were no differences between groups at baseline.

Study compliance (the self-reported consumption of study foods daily) was 81 ± 7% and 83 ± 13% for the CON and HPR groups respectively (*p* = 0.532). Both groups were advised not to change their daily exercise activity during the trial, and the physical activity (recorded as MET hours per week) did not differ between the CON and HPR groups at week 3 (43.8 ± 51.2 and 48.0 ± 33.4) or at week 6 (40.1 ± 21.7 and 46.6 ± 27.2).

**Figure 1:**
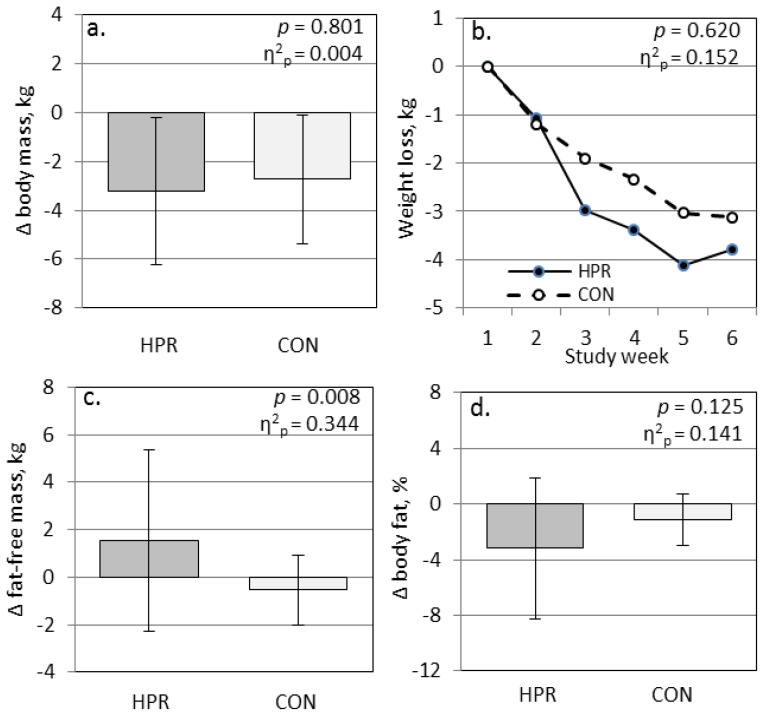
Change in (**a**) total body mass, (**b**) body mass by week, (**c**) fat-free mass, and (**d**) body fat percentage participants randomized to the energy-restricted diet composed of traditional breakfast cereal and gluten-free pasta dishes at lunch and dinner (CON; *n* = 11) or high-protein breakfast cereal and high-protein pasta dishes at lunch and dinner (HPR; *n* = 10). P represents general linear model test for change (∆) between groups controlling for gender; η^2^_p_ represents effect size (large, >0.140).

Diet record analyses indicated that participants adhered to their meal plans, and daily nutrient intakes from non-experimental foods did not vary by group (Table 3). The energy intakes for the participants that returned dietary journals (*n* = 9 and *n* = 6 for the CON and HPR groups respectively) did not differ between groups, and the average energy restriction among participants ranged from 310 to 493 kcal/day for the CON and HPR groups respectively (*p* = 0.522). The final average macronutrient composition of the CON group was 51/15/33 for carbohydrate/protein/fat as per cent of total energy, and 42/23/36 for carbohydrate/protein/fat as per cent of total energy in the HPR group. The average combined fiber intake was similar in both groups (25 g per day in the CON group and 33 g per day in the HPR group). The 6-week mean weight loss was similar for the CON and HPR groups, −2.7 ± 2.6 and −3.2 ± 3.0 kg respectively (*p* = 0.801); however, the 6-week change in fat-free mass (FFM) differed significantly between groups with CON participants losing FFM while the HPR participants increased FFM (−0.5 ± 1.5 and +1.5 ± 3.8 kg respectively; *p* = 0.008 and η^2^_p_ = 0.344) (Figure 1). This pattern in FFM change was maintained when the females were examined separately: −0.3 ± 1.3 and +3.5 ± 5.0 kg respectively. The change in body fat percentage (−1.2 ± 1.8 and −3.2 ± 5.0% respectively; Figure 1) as well as the change in fat mass (−2.2 ± 2.5 and −4.5 ± 6.4 kg respectively) did not differ significantly between groups (*p* = 0.125 and 0.158).

The 6-week change in plasma glucose and insulin, HOMA-IR, GLP-1, PYY, HMW-adiponectin, and lipids by group are shown in Table 4. Based on the 6-week reduction in HOMA-IR scores, the insulin sensitivity index improved to a greater degree for the HPR group in comparison to the CON group (−1.7 ± 1.4 and −0.7 ± 0.7 respectively; *p* = 0.020). This difference in HOMA-IR was primarily due to reductions in the fasting insulin concentrations that differed significantly between the two groups (*p* = 0.017). The change in HOMA-IR was inversely related to the change in FFM among participants (*r* = −0.511, *p* = 0.021). LDL cholesterol, triglycerides, total/HDL cholesterol, and TG/HDL cholesterol indices improved significantly over the 6-week trial among participants as a whole, but these indices did not differ between groups as a result of the intervention (Table 4). The changes in total and LDL cholesterol were related to weight loss among all participants (*r* = 0.492, *p* = 0.024 and *r* = 0.552, *p* = 0.009 respectively). The changes in the other blood indices were not related to weight change or to the change in FFM.

PYY concentrations decreased among all participants during the trial (−45% overall, *p* = 0.007); yet, the change in GLP-1 differed between groups during the trial (+0.4 ± 1.1 and −0.6 ± 0.8 pmol/L for CON and HPR respectively, *p* = 0.021). The changes in PYY and GLP-1 were correlated (*r* = 0.552, *p* = 0.012), and the change in PYY was related to the change in the TG/HDL ratio (*r* = 0.442, *p* = 0.045). Otherwise, there were no relationships between GLP-1, PYY, insulin sensitivity markers, or body composition, either at baseline or over the course of the trial. There were no differences between groups for blood markers of oxidative stress and inflammation (Table 5). Lastly, mood states were not altered by the dietary intervention (data not shown).

## 4. Discussion

Although strict calorie restriction will reduce IR, this controlled feeding trial demonstrated that the incorporation of novel high-quality protein functional food products at mealtimes with only modest calorie restriction significantly reduced IR in comparison to the use of control foods. Both sets of provided foods were similar in appearance, taste and energy content. Significant weight loss was recorded for both participant groups indicative that calorie restriction was taking place during the course of the study. However, the reduction in IR was significantly associated only with the increase of FFM. A majority of published trials have not demonstrated beneficial effects for high-protein, energy-restricted diets compared to low-protein, energy-restricted diets in terms of changes in fasting blood glucose or insulin concentrations. A meta-analysis incorporating 23 weight loss trials comparing high-protein diets against lower protein diets reported that reductions in fasting blood glucose and insulin, or in total cholesterol and LDL cholesterol, did not vary between diet groups [10]. Importantly, in these analyses the loss of FFM on energy-restricted diets was only attenuated by adherence to high dietary protein; whereas in the present report, FFM actually increased in the HPR group in comparison to the CON group, a property that likely factored into the greater success of the HPR diet for reducing IR.

Tang et al. [16] reported comparable reductions in IR in overweight men who adhered to strict, calorie-restricted diets (−750 kcal/day) for 12 weeks that were either high-protein (1.4 g/kg body weight) or high-carbohydrate, low-protein (0.8 g/kg body weight). Both groups reported similar reductions in body weight (range, 9–11 kg) and in lean body mass (range, 2–3 kg), and similar reductions in HOMA-IR scores (−1.4 and −0.9 for the high-protein and low-protein diets respectively). These investigators concluded that body weight loss factored more strongly in biomarker improvement than the quantity of dietary protein [16]. Similar results were reported for a 24-week weight loss trial incorporating both calorie-restriction (−750 kcal/day) and exercise (150 min/week) to improve IR in overweight individuals with type 2 diabetes [7]. The calorie-restricted diets were either high-protein (32% energy) or low-protein (22% energy). After 12 weeks, reductions in body weight (−8 and −7.6 kg), FFM (−1.2 and −1.8 kg), and HOMA-IR scores (−1.2 and −1.2) were similar between the high-protein and low-protein groups.

In comparison to these trials, calorie restriction was less (300 to 500 kcal) and the loss of body weight (2.7 to 3.2 kg) was modest for the 6-week trial reported herein; yet the reduction in HOMA-IR for the HPR group (−1.7) differed significantly from that for the control group (−0.7), and was greater than that noted for both the Tang et al. and Watson et al. reports. Interestingly, the protein content of the HPR diet herein was estimated at 1.4 g/kg, a level below that implemented by Tang et al. for their high-protein diet. Hence, the purposeful distribution of high-quality protein across meals (>20 g/meal) and the fact the protein was of a novel functional form may be important considerations when planning calorie-restricted diets to improve IR. Based on the work of Mamerow et al. [17], muscle protein synthesis is ~25% greater over a 24-h period when high-quality dietary protein intake is distributed evenly in daily meals (30 g per meal for breakfast, lunch, and dinner) as compared to concentrating dietary protein intake to a single meal. Additional research suggests that the effective protein dosage for muscle protein synthesis with an exercise intervention is 20 g per meal [18]. Muscle is the main site of glucose disposal, yet 25% of weight loss in calorie-restricted diets typically comes from lean mass [9]. Thus focusing on calorie restriction without attention to dietary protein distribution or the type of protein used over the course of the day may be counterproductive.

There is little research available comparing the effect of dietary protein distribution on muscle mass in healthy, non-exercising adults. In hospitalized older adults, a population likely to experience sarcopenia, Bouillanne et al. [19] reported a small but significant improvement in lean mass (+0.91 kg after 6 weeks) when dietary protein (1.5 g protein/kg/day) was distributed throughout the day versus a large intake at one meal (% distribution at 8 am, noon, 4 pm and 7 pm: 20/30/20/30 versus 6/78/2/14 respectively). Future research should explore high-protein diet therapy, with a focus on protein distribution patterns, in populations at risk of losing muscle mass, such as hospitalized patients and individuals on weight loss diets, and in populations with IR.

Participants who completed the current study were at heightened risk for metabolic syndrome based on their TG/HDL ratios, with one-third of participants displaying TG/HDL ratios above the cut-off for metabolic syndrome per group (≥3.5) [12]. Unlike the lack of difference in biomarker improvements reported for overweight adults following calorie-restricted diets with high-protein versus low-protein macronutrient distributions, individuals with metabolic syndrome following high-protein energy-restricted diets improved their metabolic risk factors when compared to lower protein energy-restricted diets. Flechtner-Mors et al. reported a 50% greater weight loss in participants with metabolic syndrome following a high-protein energy-restricted diet (1.2 g/kg) for 3 months in comparison to their counterparts following a low protein energy-restricted diet (0.6 g/kg) [20]. Furthermore, after 12 months of treatment, 65% of participants in the high-protein diet group no longer met the criteria for metabolic syndrome compared to 35% of participants in the low protein diet group. Lee et al. also reported improved outcomes for individuals with metabolic syndrome who followed a high-protein diet plan relative to those adhering to a conventional diet plan [21]. However, the results of Watson et al. suggest this benefit of a high-protein diet may be not observed in diabetic patients [7]. In the present report, weight loss was similar between groups.

The possibility of performance bias due to non-blinding of participants is a limitation of weight loss trials comparing different macronutrient compositions [16,17]. Unless high-protein and control test foods are provided in liquid or bar form, it is difficult to devise feeding trials using common food items where the participant is blinded to diet assignment. This study represented a placebo-controlled trial in which the subjects were blinded to the composition of the diet since the test foods were similar in appearance and taste; hence, performance bias was reduced. The novel high-protein functional foods utilized in this trial contained a high number of sulfur containing linkages that enabled greater cross-linking among the proteins, a property associated with a reduced rate of hydrolysis in the GI tract. In addition, this increased protein cross-linking encapsulated much of the remaining starch granules reducing their rate of absorption in the upper GI tract [22]. These factors may have impacted postprandial extrusions in glucose and insulin, possibly contributing to the improved IR noted in the present trial.

Other weight loss investigations also reported reductions in fasting PYY and GLP-1 concentrations, particularly in the early phase of energy restriction [23,24,25], and the changes in these appetite-mediating hormones may serve to encourage appetite and weight regain [26]. In some trials, high protein diets promote GLP-1 release [27,28], a characteristic believed to contribute to the satiating effect of high protein diets [29]. In contrast, the change in fasting GLP-1 over the 6-week trial was negative for the HPR group and positive for the CON group, a difference that was statistically significant. Satiety ratings were not collected from participants, but the calculated energy deficit was nearly 60% greater for the HPR group (although a non-significant difference) suggesting that the CON group did not likely have a greater level of satiety than the HPR group. The observed link between PYY and the TG/HDL ratio, a marker of risk for metabolic syndrome, is a novel finding and suggests that negative energy balance favorably influences biomarkers for metabolic syndrome.

Major study limitations include the small sample size and short treatment duration; hence, to confirm these findings longer studies with sufficient numbers of participants are needed. FFM was estimated using bioelectrical impedance technology, which may overestimate FFM as compared to the gold standard, dual-energy X-ray absorptiometry. However, the differences in these techniques are proportional across measurements and likely related to the constants incorporated into the algorithms to estimate FFM using BIA [30]. The attrition rate for this study (46%) was slightly above that reported by others [14]; however, this study had a 75% power to detect a treatment difference in HOMA-IR. Moreover, the daily dietary compliance was reportedly high (81–83%) for the participants completing the study, which suggests that the test foods were easily incorporated into the diet. The test foods were identical in appearance and taste and were provided to participants in a ready-to-eat form enhancing the internal validity of the trial.

## 5. Conclusions

This randomized controlled feeding trial demonstrated that a high protein diet (~125 g protein/day consumed in three evenly spaced meals) that include novel functional foods combined with modest calorie restriction was 140% more effective for reducing HOMA-IR in adults at risk for IR when compared to protein intake similar to a standard American diet (81 g protein/day; see [31]) in the control group with a similar level of calorie restriction. FFM increased concomitant with significant loss of body mass in the HPR participants, and this increase accounted for 26% of the improvement in HOMA-IR scores. Combining these dietary strategies (high-protein diet, even distribution of protein in 3–4 meals over the course of the day, use of novel functional foods, and modest calorie restriction) to reduce IR justifies further long-term studies, particularly given the potential of increasing FFM during calorie restriction without an increase in physical exercise.

## Figures and Tables

**Table 1:** Nutrition information for study food portions *.

	Cereal		Orzo Pasta Dish		Fusilli Pasta Dish		Total	
	CON	HPR	CON	HPR	CON	HPR	CON (% en)	HPR (% en)
Energy, kcal	260	270	324	364	324	358	935	954
Protein, g	4	21	11	25	7	24	22 (9)	70 (29)
Fat, g	5	11	9	12	9	11	23 (22)	34 (32)
Carbohydrate, g	52	24	53	39	55	39	160 (68)	92 (39)
Fiber, g	2	5	5	6	4	5	11	16

* Data for the pasta dishes represent average for 10 orzo and 10 fusilli recipes. CON: control group; HPR: high-protein group; en: energy.

**Table 2:** Baseline demographics for study participants randomized to the energy-restricted diet composed of traditional breakfast cereal and gluten-free pasta dishes at lunch and dinner (CON; *n* = 11) or high-protein breakfast cereal and high-protein pasta dishes at lunch and dinner (HPR; *n* = 10) *.

Charateristic	CON	HPR	P
Gender, M/F	1/10	5/5	
Age, years	45.6 ± 12.0	41.9 ± 12.6	0.875
Body weight, kg	86.1 ± 23.1	103.7 ± 13.9	0.310
Body mass index, kg/m^2^	30.8 ± 7.6	33.7 ± 4.7	0.561
Fat-free mass, kg	50.8 ± 12.3	64.8 ± 11.3	0.464
Body fat, %	40.0 ± 7.9	38.1 ± 11.1	0.382
TG/HDL ratio	3.5 ± 3.0	3.3 ± 1.8	0.176
Physical activity, MET h/week	41.2 ± 25.5	54.6 ± 33.8	0.649
POMS score	13.5 ± 27.4	13.3 ± 20.9	0.956

* Data are mean ± SD. P represents general linear model test controlling for gender. M/F: male/female; MET: metabolic equivalents; POMS: Profile of Mood States.

**Table 3:** Energy and nutrient intakes from non-experimental foods and total energy intake (non-experimental + experimental foods) in overweight adults following an energy-restricted diet composed of traditional breakfast cereal and gluten-free pasta dishes at lunch and dinner (CON; *n* = 9) or high-protein breakfast cereal and high-protein pasta dishes at lunch and dinner (HPR; *n* = 6) *.

Characteristic	CON	HPR	P
Energy, kcal	1184 ± 745	1206.0 ± 665	0.917
Protein, g	59 ± 43	54 ± 23	0.767
Carbohydrate, g	113 ± 70	134 ± 68	0.651
Total fat, g	55 ± 46	52 ± 41	0.765
Saturated fat, g	16 ± 10	16 ± 13	0.864
Fiber, g	14 ± 10	17 ± 10	0.628
Total energy, kcal	2119 ± 745	2160 ± 665	0.961
Energy deficit, kcal	310 ± 520	493 ± 571	0.522

* Data are mean ± SD. P represents general linear model test controlling for gender. Data represent the average of 5 days from separate weeks during the trial. Diet journals were not returned for 2 CON and 4 HPR participants. Energy intake for maintenance (Mifflin St Jeor Equation × 1.55): CON, 2429 kcal; HPR, 2653 kcal. Energy deficit represents maintenance energy minus actual energy intake.

**Table 4:** Fasting blood indices at baseline and week 6, and 6-week change data, in overweight adults on an energy-restricted diet composed of traditional breakfast cereal and gluten-free pasta dishes at lunch and dinner (CON; *n* = 11) or high-protein breakfast cereal and high-protein pasta dishes at lunch and dinner (HPR; *n* = 10) *.

Group	Baseline	Week 6	6-Week Change	P	η^2^_p_
Plasma glucose, mg/dL CON	91.0 ± 8.4	92.0 ± 9.3	1.1 ± 7.3	0.242	0.071
HPR	90.0 ± 11.4	87.6 ± 9.1	−2.4 ± 5.8		
Plasma insulin, mU/mL CON	16.7 ± 5.4	13.3 ± 4.0	−3.4 ± 2.8	0.017	0.292
HPR	22.1 ± 12.1	15.4 ± 7.9	−6.7 ± 5.0		
HOMA-IR CON	3.8 ± 1.4	3.1 ± 1.1	−0.7 ± 0.7	0.020	0.280
HPR	5.1 ± 3.4	3.5 ± 2.1	−1.7 ± 1.4		
GLP-1, pmol/L ** CON	7.3 ± 5.4	7.7 ± 6.2	0.4 ± 1.1	0.021	0.263
HPR	5.5 ± 3.0	4.8 ± 3.0	−0.6 ± 0.8		
PYY, pmol/L ^†^ CON	29.5 ± 25.1	20.6 ± 21.7	−9.0 ± 13.6	0.241	0.075
HPR	34.1 ± 30.3	14.1 ± 19.5	−20.0 ± 27.5		
HMW Adiponectin, μg/mL CON	3.0 ± 1.3	3.0 ± 1.3	+0.21 ± 2.26	0.828	0.003
HPR	1.6 ± 1.3	1.3 ± 0.9	+0.03 ± 1.16		
Total cholesterol, mg/dL CON	193.0 ± 25.7	175.5 ± 25.1	−17.5 ± 18.7	0.276	0.065
HPR	175.0 ± 19.8	149.4 ± 21.6	−25.6 ± 23.4		
LDL cholesterol, mg/dL ^†^ CON	117.4 ± 38.6	107.5 ± 29.6	−9.9 ± 19.0	0.506	0.024
HPR	108.5 ± 19.0	93.0 ± 24.3	−15.6 ± 19.3		
HDL cholesterol, mg/dL CON	49.8 ± 17.4	50.7 ± 14.2	0.9 ± 11.1	0.644	0.012
HPR	43.2 ± 12.1	43.4 ± 8.8	0.3 ± 7.3		
Triglycerides, mg/dL ^†^ CON	138.3 ± 81.0	104.3 ± 44.3	−34.1 ± 58.3	0.711	0.007
HPR	125.6 ± 49.3	83.4 ± 27.6	−42.1 ± 36.1		
Chol/HDL ratio ^†^ CON	4.3 ± 1.4	3.8 ± 1.4	−0.5 ± 0.8	0.404	0.037
HPR	4.4 ± 1.3	3.6 ± 1.0	−0.8 ± 0.7		
TG/HDL ratio ^†^ CON	3.5 ± 3.0	2.4 ± 1.6	−1.1 ± 2.1	0.882	0.001
HPR	3.3 ± 1.8	2.1 ± 1.0	−1.2 ± 1.1		

* Data are mean ± SD. Baseline values do not differ between groups (controlling for gender). P represents general linear model test for change between groups controlling for confounders (age for total and HDL cholesterol; body fat percent for insulin and HOMA); data are normally distributed with the exception of GLP-1 which was transformed for analysis. η^2^_p_ represents effect size (large, >0.140). ** one outlier removed (GLP-1: *n* = 11,9 CON,HPR). † Significanttime effect (*p* < 0.05). HOMA-IR: homeostasis model assessment-estimated insulin resistance; GLP-1: glucagon-like peptide-1; PYY: peptide YY; HMW: high-molecular weight; LDL: low-density lipoprotein; HDL: high-density lipoprotein; TG: triglyceride.

**Table 5:** Antioxidant capacity and inflammation indices in fasting blood at baseline and at week 6, and 6-week change data, in overweight adults following an energy-restricted diet composed of traditional breakfast cereal and gluten-free pasta dishes at lunch and dinner (CON; *n* = 11) or high-protein breakfast cereal and high-protein pasta dishes at lunch and dinner (HPR; *n* = 10) *.

Group	Baseline	Week 6	6-Week Change	P	η^2^_p_
Total antioxidant capacity CON	2.0 ± 0.7	2.0 ± 0.6	0.1 ± 0.5	0.410	0.036
HPR	1.9 ± 0.5	1.8 ± 0.6	−0.1 ± 0.5		
hsCRP, mg/L ** CON	3.5 ± 3.9	3.4 ± 3.5	−0.2 ± 1.2	0.533	0.022
HPR	3.0 ± 2.7	2.4 ± 1.7	−0.6 ± 1.7		
8-Isoprostane, pg/mL ** CON	18.8 ± 4.5	18.5 ± 4.8	−0.3 ± 3.8	0.909	0.001
HPR	19.0 ± 10.1	19.1 ± 3.6	0.1 ± 10.0		
TBARS, nmol/mL CON	1.7 ± 0.7	1.7 ± 0.8	−0.03 ± 1.0	0.467	0.028
HPR	2.1 ± 0.6	1.8 ± 0.4	−0.3 ± 0.6		

* Data are mean ± SD. Baseline values do not differ between groups (controlling for gender). P represents general linear model test for change between groups; data are normally distributed with the exception of total antioxidant capacity which was transformed for analysis. ƞ^2^_p_ represents effect size (large, >0.140) ** one outlier removed (8-isoprostane: *n* = 11.9 CON, HPR; hsCRP: *n* = 10 per group). hsCRP: high-sensitivity C-reactive protein; TBARS: thiobarbituric acid reactive substances.
